# RACIAL DISPARITIES IN ACCESS TO AND COST OF HEALTHCARE AMONG OLDER ADULTS WITH COGNITIVE LIMITATION: 2002 TO 2016

**DOI:** 10.1093/geroni/igz038.2085

**Published:** 2019-11-08

**Authors:** Elham Mahmoudi

**Affiliations:** University of Michigan, Department of Family Medicine, Ann Arbor, Michigan, United States

## Abstract

Using 2002-2016 Medical Expenditure Panel Survey, we examined racial/ethnic disparities in office-visits and prescription-drugs among individuals with cognitive limitation (CL). Medicare beneficiaries (65+) with CL (N=9,369) were included. We used generalized linear models. Prevalence of CL increased overtime among all racial/ethnic groups. Our findings indicate that 96% of Whites vs. 93% of Blacks had at least one office visit (diff=0.03; 95% CI:0.01-0.04). Whites had 2 (95% CI: 1.0-0.4) and 4 (95% CI: 2.5-6.0) more office visits compared with Hispanics and Asians; and used 4 (95% CI: 1-6.9), 5 (95% CI:1.0-9.3) and 6 (95% CI: 1.0-11.5) more prescriptions than their Blacks, Hispanics, and Asians, respectively. Whites had higher annual expenditures for office-visits compared with Asians ($889; 95% CI:409-1,368) and higher expenditures for prescriptions compared with Blacks ($484; 95% CI:$151-$816) and Asians ($546; 95% CI:$28-$1064), respectively. Disparities in care among older adults with CL may put vulnerable subpopulations at a higher risk.

